# Functional Assessment of Magno, Parvo and Konio-Cellular Pathways; Current State and Future Clinical Applications

**Published:** 2011-04

**Authors:** Ali Yoonessi, Ahmad Yoonessi

**Affiliations:** 1 School of Advanced Medical Technologies and Eye Research Center, Tehran University of Medical Sciences, Tehran, Iran; 2 McGill Vision Research, McGill University, Montreal, Canada

**Keywords:** Retinal Ganglion Cells, Magno, Parvo, Konio

## Abstract

The information generated by cone photoreceptors in the retina is compressed and transferred to higher processing centers through three distinct types of ganglion cells known as magno, parvo and konio cells. These ganglion cells, which travel from the retina to the lateral geniculate nucleus (LGN) and then to the primary visual cortex, have different structural and functional characteristics, and are organized in distinct layers in the LGN and the primary visual cortex. Magno cells are large, have thick axons and usually collect input from many retinal cells. Parvo cells are smaller, with fine axons and less myelin than mango cells. Konio cells are diverse small cells with wide fields of input consisting of different cells types. The three cellular pathways also differ in function. Magno cells respond rapidly to changing stimuli, while parvo cells need time to respond. The distinct patterns of structure and function in these cells have provided an opportunity for clinical assessment of their function. Functional assessment of these cells is currently used in the field of ophthalmology where frequency-doubling technology perimetry selectively assesses the function of magno cells. Evidence has accrued that the three pathways show characteristic patterns of malfunctions in multiple sclerosis, schizophrenia, Parkinson’s and Alzheimer’s diseases, and several other disorders. The combination of behavioral assessment with other techniques, such as event related potentials and functional magnetic resonance imaging, seems to bear promising future clinical applications.

## INTRODUCTION

**Figure 1:**
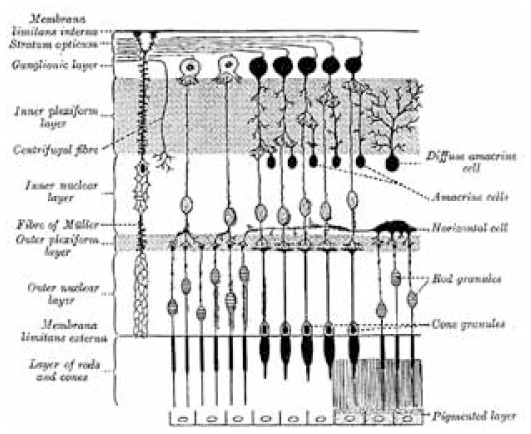
Schematic representation of the microscopic structure of the retina.

Data processing in the visual system starts in the eye itself, distinguishing it from any other organ in the body. While the eye itself is not considered as a part of the central nervous system, the multilayered structure of the retina enables early processing of retinal cone responses. Cone photoreceptors consist of three cell types known as long, medium, and short (L, M, and S), are located in the outermost layer of the retina, and are connected to the ganglion cells by bipolar cells. A complex interconnected network is created with horizontal cells located between cones and bipolar cells, and amacrine cells in between bipolar and ganglion cells (Fig. 1).

## RETINAL GANGLION CELLS

Ganglion cell axons travel from the retina to the lateral geniculate nucleus (LGN) and then to different parts of the visual cortex. Several physiologically and morphologically distinct types of ganglion cells exist. One, known as the magno cell, responds rapidly to stimulation, has thick axons with more myelin and large receptive fields (i.e., collects information from several cones). Another type known as the parvo cell, has thin axons with less myelin, responds slowly to stimuli and has smaller receptive fields. In the LGN, distinct layers of magno and parvo cells can be identified using several staining methods. Magno cells collect information from all types of cones; hence they detect “luminance” and can signal motion, stereopsis and depth. The parvo system is mostly used for detection of “chromatic” modulation, and thus the form and material of an object. Their processing pathways also differ: magno cell information is processed through the “where” pathway to the parieto-occipital cortex, while information from parvo cells is mostly processed through the “what” pathway in the inferior temporo-occipital cortex.^1^

One other major type of ganglion cells is known as the konio cell. Konio cells are much less in number than the other two types and form three tiny separate layers in between the magno and parvo cells in the LGN. Structurally, they are smaller than parvo cells. The physiological response of konio cells is not as well studied as the other two cell types, while they may play a role in seasonal mood changes^2^ and color constancy mechanism^3^,^4^. Seasonal mood changes might as well be affected by the recently-found ganglion cells known as melanopsin containing retinal ganglion cells (mRGC).^5^

Evidence has accumulated that these three pathways show characteristic patterns of malfunction in multiple sclerosis (MS)^6^,^7^, schizophrenia^8^,^9^, Parksinson’s^10^ and Alzheimer’s disease (AD)^11^,^12^, and several other disorders^13^,^14^. The combination of behavioral assessment and other techniques, such as event related potentials^15^ and functional magnetic resonance imaging (fMRI)^16^,^17^, has been shown to bear promising future clinical applications.

It should be noted that about 7 to 12 other types of ganglion cells, including a photosensitive ganglion cell^5^, have been reported.^18^ However, these cell types are much less frequent and do not create distinct layers in the LGN.

## RECEPTIVE FIELDS

Bipolar cells collect, compare, and relay information from cone photoreceptors to ganglion cells. Complex networks of horizontal and amacrine cells assist in the collection and comparison of information received from cones. Cone photoreceptors that provide input to a ganglion cell through bipolar cells are called the receptive field of that particular ganglion cell.

Two types of bipolar cells have been identified. The first polarizes in the same way that cones polarize, i.e., hyperpolarization in reaction to light; these are called “Off” cells. The other type, the “On” cells, polarizes in the reverse direction. This difference arises from different glutamate receptors on the surface of these cells. In the fovea, where visual acuity is highest, most cones connect with one “Off” and one “On” bipolar cell.^18^,^19^ In the periphery, the number of On/Off cells increases. Overall, such a structure creates a receptive field, which, for example, can detect an edge or create a center/ surround organization.^20^

## TRANSFER OF INFORMATION FROM THE RETINA TO THE VISUAL CORTEX

The distinctive structural, morphological and physiological features of the three types of ganglion cells have made them a good candidate for early diagnosis and treatment of several diseases. For example, evidence exists that the demyelination process in multiple sclerosis affects magno and parvo pathways, in a specific order.^1^ In addition to distinct features of the three pathways, information compression which occurs in the optic nerve facilitates the detection of any ganglion cell pathology. The optic nerve, with its 1.6 million fibers, carries information from about 4 million cones.^21^ Therefore, data compression occurs at the retinal level. In a model of L, M, and S cone responses in natural environments, a mathematically optimized solution for data compression matches the physiological response of the three types of ganglion cells.^22^ A mathematical model frequently used in the engineering field, known as “principal component analysis”, can extract the principal components of data. With linear transformation of data, the axes rotate to match the principal components such that the smallest number of “codes” is required to transfer information between two points. The principal components of a model of LMS cone responses match the function of magno, parvo, and konio cells, i.e., the first principal component detects only luminance (similar to magno cells), the second detects a comparison of L and M cones (similar to parvo cells), and the third matches a comparison between S and the average of L and M cone responses (similar to konio cells).

A similar mathematical model, known as independent component analysis, yields filters (or small templates) similar to simple cells in the visual cortex, where the ganglion cells synapse.^23^ The extraction of filters in the cortex relies on the structure of the receptive fields in early layers, including ganglion cells.

“Data decompression” seems to take place in the cortex, since the 1.5 million ganglion cells connect to about 120 million neurons in the visual cortex.^24^ Therefore, lesions in each of the three compressed pathways cause considerable functional loss as compared to lesions in other areas of the central nervous system. This fact has been used in clinical practice since long ago; for detection of tumors that may compress the optic nerve and lead to signs such as reduced visual acuity, visual field defects, and optic atrophy or edema. Advances in technology have enabled researchers to detect subtle changes in a variety of other disorders, in addition to optic nerve tumors.

## METHODS OF ASSESSMENT OF GANGLION CELL FUNCTION

Most of the available information on retinal ganglion cells originates from animal models. In animals, electrophysiology has been the main method for assessing ganglion cell function. Recording electrical activity can be performed at the intracellular level, the extracellular level, or using multiple electrodes with tens or even hundreds of concurrent activity recordings. Most data comes from studies on pigeons, owls, cats, and monkeys, particularly macaque and rhesus monkeys which are assumed to be very similar to humans. The ability of monkeys to be trained has provided extensive insight by combining electrophysiological methods with behavioral, imaging, and histological methods.

Noninvasive methods used in humans include behavioral and psychophysical techniques combined with functional imaging, such as fMRI, PET (positron emission tomography) scan and NIRS (near infrared spectroscopy). Noninvasive electrophysiological data from humans using electroencephalography (EEG) or EEG during presentation of stimuli, known as event-related potentials (ERP), have been valuable for evaluation of ganglion cell function. Therapeutic interventions for epilepsy and Parkinson’s disease have sometimes provided the opportunity to record invasively from humans.^25^

Behavioral techniques include simple tasks, such as contrast sensitivity measurement, designed to distinctively stimulate magno, parvo, or konio cells. Stimuli containing only luminance information (i.e., with no chromatic modulation) selectively stimulate magno cells, while chromatic modulation without any luminance changes elicits a response from parvo and konio cells. Since parvo cells mainly receive input from L and M cones in an excitatory/ inhibitory combination, a combination of chromatic stimuli that selectively excite L and M cones can excite parvo cells. Such stimuli may consist of two colors close to dark red and dark green with the same luminance levels (isoluminant). Konio cells, on the other hand, seem to compare inputs from S cones with either L or M cone inputs. Therefore, an isoluminant stimulus combining violet/blue with greenish yellow would selectively provoke a response from konio cells. More complex tasks have been used to determine the function of each of the pathways in more detail. For example, by decomposition of natural images to information that selectively excites magno, parvo, and konio cells, it has been shown that the structure of natural scenes plays a major role in ganglion cell function, and this role is much more prominent for parvo cells as compared to magno cells.^4^

Behavioral techniques have been used in combination with EEG recordings as well as functional imaging methods. For example, during visual evoked potential (VEP, the event related potential generated by visual stimuli), a C1 component was identified. C1 is a positive signal recorded in the central parieto-occipital area, beginning 40 to 60 ms after the stimuli is shown and reaching its peak at around 70 to 100 ms when it resolves into a P1. C1 has been reported to be present when only parvo cells are excited (chromatic isoluminant stimuli), but not with luminance-only stimuli (magno cells).^26^ It has been possible to extract other meaningful information from ERP as well. In an interesting study, the timing of display of a target stimulus among some non-target stimuli could be identified by a single trial.^27^

Numerous studies have used retinotopic maps in combination with fMRI to locate the cortical area responsible for processing chromatic or luminance information. One study has reported that the superior colliculus, which receives a tiny part of information from the retina, was mainly activated by luminance stimuli only and not by other chromatically modulated stimuli.^16^ The low temporal resolution of functional imaging methods has been a major obstacle for their use in combination with behavioral tasks.

## CLINICAL FINDINGS

### Glaucoma

In ophthalmology, two recent perimetry methods are based on evaluation of selective cone/ ganglion cell functions using psychophysical tasks. Frequency doubling technology (FDT) perimetry was launched in 1997 and since then, several studies have shown its sensitivity for detection of early glaucoma and other disorders.^28^–^30^ The device is based on the perception of an illusion requiring intact function of the magnocellular pathway. In this illusion, when an achromatic sinusoidal low-spatial frequency grating is flickered in counter-phase at frequencies above 7 Hz, the spatial frequency seems to be doubled. Since only magno cells can respond rapidly to flickering stimuli, inability to elicit this illusion implies magno cell dysfunction, a situation that occurs in early glaucoma, ocular hypertension^31^, and even HIV infection^32^. Shortwave automated perimetry (SWAP) utilizes a bright yellow background with superimposed blue stimuli that specifically evaluates the function of S cones, and hence konio cells.

A recent comparison of visual evoked potentials of cone photoreceptors demonstrated that the S cone response in patients with open angle glaucoma had a different slope for the first positive wave as compared to age-matched healthy subjects. However, no difference in L or M cone responses were observed.^33^

### Multiple Sclerosis

Multiple sclerosis has been a subject of intense research in the past decade. While uveitis and periphlebitis have already been recognized as ophthalmologic findings in MS, other findings such as nerve fiber layer changes have recently been under scrutiny. Optical coherence tomography demonstrated thinning of the retinal nerve fiber layer in multiple sclerosis which surprisingly did not correlate with optic neuritis.^34^–^36^ A postmortem analysis of 82 MS patients has shown cone and ganglion cell loss in 79% of cases and a loss in amacrine and bipolar cells in 40%.^37^ In another study, 8 postmortem MS patients showed a significant loss of parvo cells in the LGN.^38^ The results of psychophysical tests have been controversial. In MS patients with optic neuritis, parvo cells seem to be affected based on tests including luminance-modulated^39^ and chromatic-modulated^40^ contrast sensitivity, visual evoked potentials^41^ and temporal frequency discrimination^42^. In early MS cases with no optic neuritis, patients are almost equally divided into three groups: damage limited to magno cells, parvo cells, or both.^6^ Contrast sensitivity impairment at high spatial frequencies was suggested to be indicative of parvo cell damage, while low spatial frequency impairments were associated with magno cell damage. In the same study, N1 and P1 VEP responses were correlated with parvo and magno cell responses, respectively. Psychophysical data were supported by impairment in VEP responses which occurred in addition to spatial frequency contrast sensitivity impairments. Murav’eva et al^6^ suggested that MS patients who develop visual symptoms have parvo cell damage, while those with magnocellular layer damage are usually asymptomatic.

### Alzheimer’s Disease

Evidence suggests rapid deterioration of the magnocellular pathway in Alzheimer’s disease. AD patients tend to underreport their visual problems in comparison with healthy age matched controls, therefore their visual problems are left unnoticed. Eight out of ten AD patients were reported to have extensive axonal degeneration in the optic nerve^43^ and these findings were supported by follow-up studies^44^,^45^, though other studies disagree with these findings.^46^,^47^ This is in contrast to the widespread belief that AD does not involve sensory perception. Moreover, postmortem biopsies of the LGN demonstrated plaques in both parvo and magno layers; interestingly parvo layers showed a higher density of plaques.^48^

Psychophysical tasks of motion coherence and flicker detection, which evaluate the function of magno cells, show significant functional loss in AD patients which increases with the severity of the disease.^49^,^50^ VEP tasks using chromatic and achromatic modulation have revealed delays with achromatic stimuli only, confirming magnocellular pathway involvement in AD.^12^

### Schizophrenia

Most of the deficits found in schizophrenia have been reported to involve magno cells, however parvo cells seem to be affected as well. Abnormalities in motion perception and sensitivity to low contrast achromatic stimuli have been reported.^51^ However, chromatic stimuli that selectively elicit a response from parvo cells have also shown deficits in schizophrenic patients.^52^ VEP responses of schizophrenic patients have shown a lower amplitude compared to normal subjects for luminance modulated stimuli (magno cells), but not for chromatic modulated stimuli (parvo cells).^53^,^54^

### Other Disorders

Severe magno cell deficits have been reported in patients with Parkinson’s disease and are correlated with the stage of the disease. Parvo cells seem to be affected as well, but not in correlation with disease severity and independent of magno cell damage. Konio cells have been reported to be intact in Parkinson’s disease.^10^

Tests for magno and parvo cell function have been reported to be impaired in early chiasmal tumors.^14^ Magno cell deficits have also been suggested to be involved in migraine.^55^ Dyslexia is another disorder for which the magno cells seem to be more involved than the other two pathways.

## CONCLUSION

Recent advances in techniques of selective stimulation of the three different ganglion cell pathways have revealed specific deficits in various disorders. Assessment of each of these three pathways can therefore be used to diagnose or monitor these impairments. The combination of behavioral techniques with electrophysiological and imaging methods has been able to delineate specific damage in each pathway.

The compression of data that occurs during transfer of data from the retina to the cortex makes the pathways very sensitive to various types of damage and a good candidate from a diagnostic point of view. Recent advances have shown important clinical utilities for such tests which need to be thoroughly examined. Easy access to the eyes, simple and independent excitation of each of these pathways, and the possibility of combination with other techniques, promises clinical applications for such tests.

Several common disorders in ophthalmology, such as ocular hypertension and glaucoma, show distinctive patterns of damage to retinal ganglion pathways. Neurological disorders that affect retinal ganglionic pathways include MS, Alzheimer’s disease, and Parkinson’s disease. Some psychiatric disorders such as schizophrenia and depression have been shown to affect retinal ganglion pathways in a similar way.

A potential diagnostic device that would evaluate all three pathways with simple functional tests and combine behavioral tests with simultaneous EEG recordings, could be an essential part of many clinical settings in the near future.
